# Comparison of the Foamability of Linear and Long-Chain Branched Polypropylene—The Legend of Strain-Hardening as a Requirement for Good Foamability

**DOI:** 10.3390/polym12030725

**Published:** 2020-03-24

**Authors:** Nick Weingart, Daniel Raps, Mingfu Lu, Lukas Endner, Volker Altstädt

**Affiliations:** 1 Department of Polymer Engineering, University of Bayreuth, 95447 Bayreuth, Germany; nick.weingart@uni-bayreuth.de (N.W.); daniel.raps@gmx.net (D.R.); endner.lukas@gmx.de (L.E.); 2 SINOPEC Beijing Research Institute of Chemical Industry, Beijing, 100013, China; lumf.bjhy@sinopec.com

**Keywords:** polypropylene, foam-extrusion, morphology, foaming, crystallization kinetics

## Abstract

Polypropylene (PP) is an outstanding material for polymeric foams due to its favorable mechanical and chemical properties. However, its low melt strength and fast crystallization result in unfavorable foaming properties. Long-chain branching of PP is regarded as a game changer in foaming due to the introduction of strain hardening, which stabilizes the foam morphology. In this work, a thorough characterization with respect to rheology and crystallization characteristics of a linear PP, a PP/PE-block co-polymer, and a long-chain branched PP are conducted. Using these results, the processing window in foam-extrusion trials with CO_2_ and finally the foam properties are explained. Although only LCB-PP exhibits strain hardening, it neither provide the broadest foaming window nor the best foam quality. Therefore, multiwave experiments were conducted to study the gelation due to crystallization and its influence on foaming. Here, linear PP exhibited a gel-like behavior over a broad time frame, whereas the other two froze quickly. Thus, apart from strain hardening, the crystallization behavior/crystallization kinetics is of utmost importance for foaming in terms of a broad processing window, low-density, and good morphology. Therefore, the question arises, whether strain hardening is really essential for low density foams with a good cellular morphology.

## 1. Introduction

Polypropylene (PP) is an outstanding choice as matrix material for polymeric foams due to its favorable mechanical properties and chemical resistance. However, its low melt strength and fast crystallization result in unfavorable foaming properties. It is difficult to obtain homogeneous lightweight foams due to their low melt elasticity, viscosity, and low temperature dependence of melt viscosity in fully molten state, which makes the control of viscosity by temperature variation highly challenging. Furthermore, crystallization can occur during foaming, which can lead to either shutdowns of the process (freezing of the die) or unfavorable foam structures due to cell rupture. It is concluded, that a good knowledge on the rheology and crystallization properties is essential to adjust the morphology and thereby the elastic modulus, strength [1], impact behavior [2], and thermal conductivity [3] of foamed products. 

During foam extrusion, temperature, pressure, and blowing agent affect the flow behavior of the melt and, ultimately, the foam properties. In addition, the melt behaves differently under shear and elongational flow. The flow patterns in the extruder are dominated by shear deformation. However, the elongational properties become relevant after the melt leaves the die, as in the foaming stage, the melt is subjected to elongational deformation during bubble growth, namely, equi-biaxial extension. The rheology of polypropylene is a major challenge in achieving low-density foams. Generally, PP has a comparatively low viscosity in fully molten state compared to amorphous polymers such as polystyrene or polycarbonate.

For foaming semicrystalline polymers like PP, the crystallization behavior is one of the key factors for good and controllable foamability. In the state-of-the-art for continuous foaming, the polymer is first molten, mixed with the blowing agent, followed by subsequent cooling of the melt/gas solution along with increasing pressure towards the die. Thus, the temperature of the melt at the die (relative to the onset of crystallization) determines how long the polymer remains in the molten state until it solidifies. For semicrystalline polymers, this threshold value is the crystallization temperature, which can be shifted towards lower values due to the plasticizing effect of the blowing agent (concentration-dependent) [4]. As soon as the expanded polymer melt reaches this temperature, the foam starts solidifying. The outer shell solidifies first as it is exposed to the cold surrounding. In the foam core, the higher temperature is retained longer, which can cause cell/foam coalescence if the favorable foam structure is not stabilized (frozen) in time. Therefore, it is a necessity to cool down the melt to an optimal temperature window (processing window). A temperature that is too low causes a very high viscosity (freezing of the die for semicrystalline polymers) and inhibits the growth of the foam cells, whereas a high temperature makes it difficult to stabilize the foam (low viscosity and high solidification time) to keep the blowing agent dissolved, and finally leads to foam collapse. Conclusively, the processing window represents a compromise between remaining temperature (required for elasticity, expansion, and stabilization) and foaming potential/time (crystallization speed and blowing agent concentration) [5,6,7,8,9,10,11,12,13]. Therefore, for standard grades of polypropylene, the temperature window for extrusion foaming is generally very narrow.

Furthermore, the elongational properties of the melt are of utmost importance, as they govern the cell growth and influence the rupture of the forming cell walls. The viscoelastic behavior of the melt in elongational deformation is crucial for the formation of the foam morphology [14], as mostly low average cell size, a narrow cell size distribution, and low density are required. Polypropylene is well known to possess very disadvantageous elongational properties; both melt strength and drawability are very low for standard grades.

Fortunately, chain topology can be modified in order to improve its foamability. In particular, the effect of strain hardening at large elongational strains is desired to support cell stabilization. In this frame, strain hardening is highly beneficial for foaming due to the effect of self-healing of the cell walls during the expansion of foam cells. Strain hardening means the rise of the elongational viscosity above the zero-rate elongational viscosity. The strain hardening helps to prevent cell coalescence and to widen the processing window [13], i.e., for the foaming of PP [15]. In terms of chain topology, strain hardening is caused by long-chain branching (LCB) [7,8]. Furthermore, LCB often causes thermo-rheological complexity, as shown by several authors [16,17,18]. Although the determination of thermo-rheological complexity is not straightforward, it can help to widen the temperature processing window. For example, Raps at al. [19] found that long-chain branching leads to an increased temperature sensitivity of the melt viscosity at strain rates relevant for foaming, which should allow a better process control during foaming. However, LCB-PP has the disadvantage of a lower solubility of CO_2_ compared to linear PP [20]. This arises due to the fact that LCB-PP has a lower specific volume compared to linear PP as well as a more pronounced resistance against swelling, which is caused by dissolution of CO_2_ [21].

The crystallization kinetics and crystalline morphology of ready available semicrystalline polymers are well understood because of their high importance for the manufacturing of polymer products by techniques like fiber-spinning, blow molding, and injection molding [22]. In the case of foam and cellular polymeric materials, although to a lesser extent, it is well known that crystallization phenomena play a major role in foaming [23]. Crystallization phenomena at the relevant conditions for gas loaded polymers intended for foaming must be understood to obtain good foam morphologies, to reduce development time and set the lower limit of the processing window of temperature. Many factors change the crystallization behavior of polymer melts. Those factors will be discussed subsequently.

The effect of pressure on the crystallization of various polymers has been discussed in many publications in the past. Elevated pressure leads to higher crystallization temperatures as the driving force for chain alignment is increased. Besides pressure and cooling rate, also the molecular structure determines the crystallization behavior. Especially important is long-chain branching (LCB), as discussed before. One effect of LCB on crystallization is an increase in crystallization temperature [24] and the amount of γ-phase [11].

As gas is dissolved into the polymer for the purpose of foaming, its effect on crystallization must be considered as well. Takada et al. studied the effect of CO_2_ on crystallization of iPP [25]. Dissolved gas increases the molecular mobility and the free volume, thus leading a decrease of chain–chain interactions. Because of this the motion of chains into the crystal-amorphous boundary is improved, leading to a higher crystallization rate. It can be summarized that, if crystallization is nucleation-controlled, the overall rate is decreased by the incorporation of CO_2_; otherwise, it is increased. Takada et al. also investigated the effect of dissolved CO_2_ on the isothermal crystallization of PET in yet another work [26]. They found that if the reduction of the glass transition temperature T_g_ is higher than the melting temperature T_m_, the crystallization rate is increased. If their reduction is fairly similar, the crystallization rate is decreased (nucleation-controlled region). They concluded that this rule may be applicable to all semicrystalline polymers. Moreover, Oda and Saito [27] found that spherulite growth rate reached an optimum at moderate CO_2_ pressure, thus emphasizing the competition between plasticization effects, which dominate at low concentrations, and exclusion effects pushing CO_2_ away from the crystallization front at high CO_2_ concentrations. However, there is also a back side in crystallization: the occurrence of crystals leads to a significant reduction in gas solubility as well as diffusivity [23].

Furthermore, the timing and kinetics of crystallization are crucial for a well-defined and fine cellular morphology. If crystallization proceeds too quickly and in a very narrow window, the danger of the die freezing in foam extrusion is more pronounced. Also, if crystallization takes place during cell growth, crystals contribute as a nucleus for bubble growth, but it might also result in a collapse of the cellular structure, become very inhomogeneous or partially open-cellular. Moreover, effects like nucleation due to shear and elongational flow must be taken into account [28]. 

It can be seen that both rheology and crystallization play an important role in foaming. Especially, long-chain branching is regarded as a major contributor for good foam morphology, i.e., low density and small cells with a narrow distribution by causing strain hardening. Currently, strain hardening is generally regarded as a prerequisite for good foamability of semicrystalline thermoplastics. However, is strain hardening really essential for low density foams with a good cellular morphology? This paper aims to shed some light on this question and whether other factors might also come into play. Therefore, we study the rheology and crystallization properties of three PPs (linear PP with a broad molecular weight distribution, a PP-PE block-co-polymer, and a LCB-PP as benchmark material).

## 2. Materials and Methods 

In the scope of this work, three polypropylene grades were studied: two PP grades of HMS20Z (linear, homopolymer) and E02ES (linear, PP-PE copolymer) were provided by Sinopec, 100013 Beijing, China, (trade names) as high melt strength PP. They will be referred to as Sinopec HMS20Z and Sinopec E02ES CoPo, respectively. Sinopec HMS20Z has a M_W_ of 474,000 g/mol (PDI of 11.05) and a MVR of 2.1 g/10 min (230 °C with 2.16 kg). The higher melt strength of the materials is achieved through a broad molecular weight distribution. Sinopec E02ES CoPo has a M_W_ of 456,000 g/mol (PD of 9.16) and a MVR of 1.5 g/10 min (230 °C with 2.16 kg). The third HMS-PP grade, Borealis Daploy WB140HMS with a M_W_ of 350,000 g/mol (PDI of 4.6) and a MVR of 2.1 g/10 min (230 °C with 2.16 kg), was used as a reference in this work (referred to as Borealis WB140 HMS). The higher melt strength of this grade is generated through long-chain branching. The Sinopec materials are chemically pure. Borealis WB140 HMS is a commercial grade, thus additives cannot be excluded. 

The determination of the crystallization kinetics was performed with a Mettler Toledo DSC 1, 43085 Columbus, OH, USA (cooling realized by a compressor) in the temperature range of 0 to 220 °C with a heating rate of 10 K/min and cooling rates of 2, 4, 8, 10, and 16 K/min under nitrogen atmosphere. The evaluation was carried out with the STARe-software with a Δ H_m_^0^ of 207.1 J/g [29] according to Khanna et al. [30] with a straight base line. 

The analysis of the non-isothermal crystallization kinetics for the DSC results was performed according to Jeziorny modified Avrami theory [31]. The DSC thermograms are often inconclusive where the crystallization process begins and where it ends. Avrami plots strongly depend on the shape and position of the borders of the crystallization process. As no clear reproducible proceeding was reported in literature, and to guarantee reproducibility, the heat flow was differentiated (1^st^ order) and plotted over temperature to identify the start and the end of the crystallization window for the Avrami evaluation. 

The rheological investigation in shear deformation was performed with an Anton Paar MCR 702 TwinDrive, 804x Graz, Austria rotational rheometer under nitrogen atmosphere (50 mL/min) with specimen geometry of 25 mm diameter and 2 mm thickness. Strain sweeps were carried out in the deformation range of 0 to 100% with an angular frequency of 1 rad/s at 180, 190, and 200 °C. Frequency sweeps were performed at constant temperature of 180, 190, and 200 °C with a decreasing angular frequency of 200 to 0.1 rad/s and amplitude of 5 %. Non-isothermal multiwave (NiMW) measurements were performed with an Anton Paar MCR 702 rotational rheometer under nitrogen atmosphere and cooling rates of 0.5, 1, 2, and 4 K/min from 200 to 100 °C to determine the gel point due to crystallization and the starting point of the crystallization. The harmonics were chosen as factor 5, 25, and 125 of the fundamental sinus wave (1 rad/s).

Isothermal multiwave analysis was analogously performed with an Anton Paar MCR 702 TwinDrive device under nitrogen atmosphere. The measurement procedure for this work was specifically designed to prevent the specimen from premature crystallization. The measurement procedure consisted of three steps: (**I**) Cooling the polymer melt to a temperature 1 °C above the investigation temperature (previously determined by DSC), while the shear stress is increased from 25 to 40 Pa. (**II**) Cooling down from the onset temperature to the investigation temperature with constant shear stress of 50 Pa. (**III**) Measurement at investigation temperature with 1 rad/s and a shear stress of 50 Pa. Sinopec E02ES CoPo was investigated at 130, 129, 128, and 127 °C; Sinopec HMS20Z at 136.5, 135.5, 134.5, and 133.5 °C; and Borealis WB140 HMS at 148.5, 147.5, 146.5, and 145.5 °C. The normal force was kept constant at 0 N during the measurements as the specimen volume decreases due to crystallization. In Table 1, the measurement procedures are exemplarily displayed for Sinopec HMS20Z.

The investigation of the melt strength of the PP-grades was performed with a Rheotens 71.97 mounted on a Göttfert high-pressure capillary rheometer 6000, 74722 Buchen, Germany. Previous to testing, the material was dried overnight at 70 °C under vacuum. The measurement was carried out at a melt temperature of 220 °C and a shear rate of 30 s^−1^. The temperature was carefully chosen to avoid strand expansion and sagging of the melt after leaving the die, hence preventing internal stress. The nozzle diameter was 2 mm with a length of 30 mm and a distance of 95 mm between the nozzle and the upper wheel. The maximum wheel speed (measuring range) was set to 1000 mm/s, with a starting speed of 7.5 mm/s.

Furthermore, the elongational viscosity was studied using a universal extensional fixture (UXF). This is a filament stretching tool for rotational rheometers. The device was manufactured by Anton Paar, Austria and used with a MCR 702 rheometer. A strip-like sample of 10 × 18 × 0.6 mm^3^ is uniaxially stretched by means of two cylinders, which rotate around their own axis (ω1 = ω2). The tensile stress is obtained from the measured torque and the strain rate from the rotational speed. The maximum Hencky strain $\varepsilon =\ln\left(\frac{l_{max}}{l_{0}}\right)$ is 5 for one revolution of the drums. To reduce the sagging of the sample, it was subjected to a pre-stretch. Therefore, a constant torque of 2.5 μNm at 180 °C, which equals a stress of 81 Pa in the sample, and 1.25 μNm at 200 °C, which equals a stress of 47 Pa in the sample, was applied. As a drawback, this can cause measuring errors due to a certain degree of orientation of the polymer chains. However, the error due to this initial tension on the results of the elongational experiments is markedly smaller than that of a deformed sample due to gravity. The final stretching step was carried out at strain rates of 1, 3, and 10 s^−1^ up to a Hencky strain of 4.7. The higher elongation rates were deliberately chosen to simulate the rates occurring during foaming [13].

The foam extrusion was carried out on a tandem extrusion line from Dr. Collin GmbH. The line consists of a twin-screw kneader ZK 25 P x 42 L/D, maximum throughput of 15 kg/h and (A-extruder) with co-rotating screws for compounding and gas injection and a single-screw extruder E 45 M x 30 D (B extruder) for pressure build-up and cooling. The foam extrusion line was operated at a throughput of 5.5 kg/h, 2–6 wt.% CO_2_ as blowing agent (supercritical dosing by a Maximator) and a nozzle temperature of 190 °C (156 °C for Sinopec). The nozzle diameter was 3 mm and the length 100 mm. The speeds of rotation for the screws were 135 rpm in the A-extruder and 12 rpm (9 rpm for WB140HMS) in the B-extruder. The single-screw in the B-Extruder has a constant flight depth over the whole screw. The sealing applied to prevent CO_2_-loss works via a melt buffer upstream of the transfer pipe between A- and B-Extruder. 

The aim was to determine a processing window for foam extrusion, defined through best foam properties in regard to density and morphology, for the investigated PP-grades. Foaming Borealis WB140 HMS was rather challenging, as high pressures and viscosities narrowed the operating window. As well, this grade was not able to contain more than 4 wt.% of CO_2_ (more blowing agent resulted in strong fluctuations and gaseous leakage at the die). For general comparison, the materials with the best result were chosen. Detailed processing parameters and the found processing windows are summarized in Results. No additives were used for comparable neat material characterization.

The foam morphology was studied with a scanning electron microscope JEOL JSM-6510, 196-8558 Tokyo, Japan with an acceleration voltage of 10 kV and a secondary electrons (SE) detector. The samples were fractured in liquid nitrogen and sputtered with a 13 nm thick gold layer. Evaluation of cell diameters and cell size distribution was performed with the program ImageJ on an average of at least 80 cells.

## 3. Results and Discussion

In the following the characterization of the samples is laid out. Thereafter, the results of the foam extrusion trials (processing window and the final foam morphology) are presented and explained with the characteristic properties of the PPs.

### 3.1. Characterization of the Base-PPs

#### 3.1.1. Characterization of the Crystallization Behavior

For the characterization of the thermal properties, heating and cooling rates of maximum 16 K/min were used. During the actual foaming process, the melt leaves the die at the temperature between T_die_ and T_melt_ and is cooled to ~50 °C usually in less than one minute. Those high cooling rates cannot be realized with standard DSC measurements and therefore have to be assumed, based on lower possible cooling/heating rates analog to literature. Nevertheless, it gives good insight into the crystallization behavior and how the crystallization behavior progresses with increasing cooling rates [31,32].

##### Non-Isothermal Crystallization Kinetics

**Figure 1:**
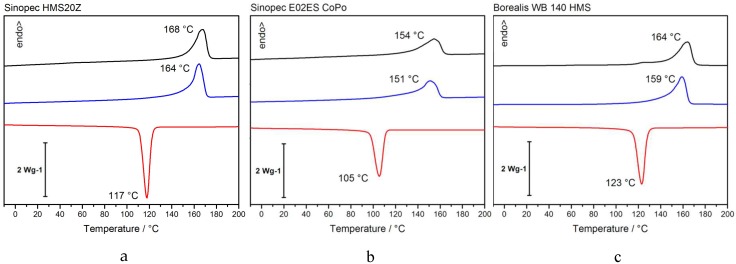
DSC-analysis of Sinopec HMS20Z (**a**), E02ES CoPo (**b**), and Borealis WB140 HMS (**c**) at 10 K/min.

First, thermal characterization of the different PP-grades is presented. The three samples differ in their total crystallinity (Figure 1; Table 2) as well as the melting/crystallization peak temperature. The Sinopec HMS20Z has the highest degree of crystallinity with 54%, followed by Borealis WB10 HMS with 44% and finally Sinopec E02ES CoPo with 40%, showing that a small amount of PE in the backbone reduces crystallinity more than long chain branches.

**Figure 2:**
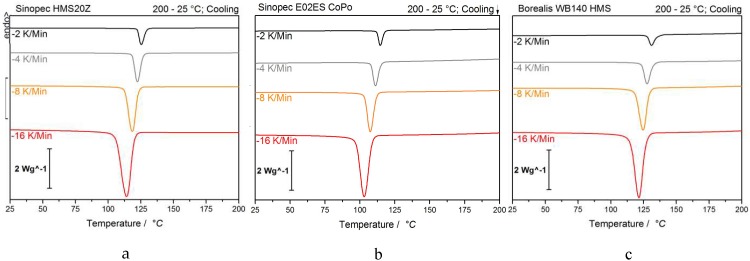
2^ND^ cooling curves of Sinopec HMS20Z (**a**), E02ES CoPo (**b**), and Borealis WB 140 HMS (**c**) at −2, −4, −8, and −16 K/min used for Avrami evaluation.

The determination of the half time point for crystallization and the kinetic study samples was measured at different cooling rates. As depicted in Figure 2, the temperature range for crystallization expectedly shifts towards lower temperatures for higher cooling rates and the higher supercooling in all samples. 

To compare the non-isothermal crystallization kinetics (in dependence on the cooling rate) of the PP grades with multiwave measurements, an evaluation with the Avrami approach was performed:

(1) $$X_{t}=1-\exp\left(-k\;t^{n}\right)$$

(2) $$\ln\left[-\ln\left(1-X_{t}\right)\right]=\ln k+n\ln t$$

The Avrami evaluation is used to describe isothermal crystallization kinetics with *X_t_* being the time-dependent relative crystallinity, *k* is a constant representing the crystallization rate, and *n* is the Avrami-exponent describing the crystal growth and the crystalline superstructures. However, the Avrami-approach ignores the influence of cooling rate and thermal gradient on the sample. Thus, to describe the non-isothermal crystallization kinetic, a modified Avrami theory by Jeziorny [31] was used. This approach considers the heating rates φ, as they influence the nucleation and growth of the crystalline polymer phase. The constant for the kinetic crystallization rate *k_c_* is described as follows,

(3) lnkc=lnkφ

**Figure 3:**
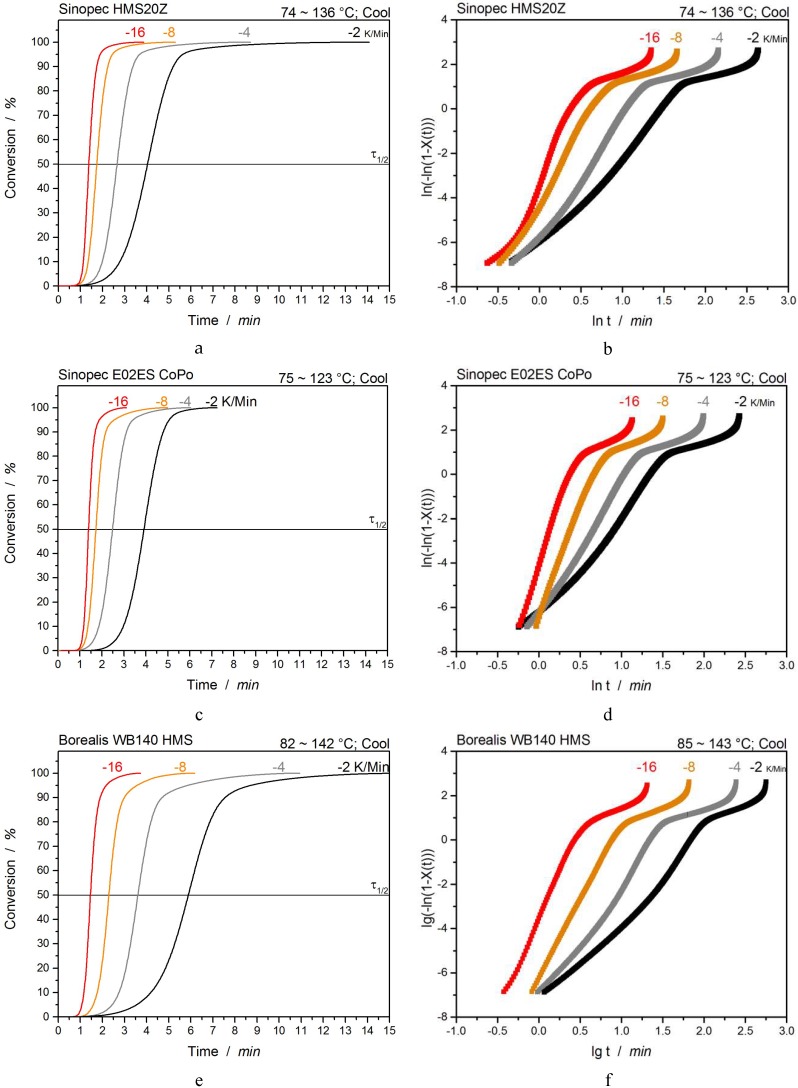
Conversion- and Avrami-plots of Sinopec HMS20Z (**a**,**b**), Sinopec E02ES CoPo (**c**,**d**), and Borealis WB 140 HMS (**e**,**f**).

In Figure 3, the conversion plots of each PP grade over time are shown beside the corresponding Avrami–Jeziorny plots. Both Sinopec grades show a similar course of conversion with comparable times for the half-time crystallization τ_1/2_, whereas Borealis WB 140 HMS takes longer times (at lower cooling rates) to reach 50% crystallinity. At higher cooling rates, the differences become more and more negligible. Additionally, it is evident that the whole crystallization process for Sinopec E02ES CoPo is very fast and finishes after ~7 min, whereas Sinopec HMS20Z takes twice as long and Borealis WB140 HMS takes even longer. Detailed values are listed in Table 2.

For low cooling rates, all Avrami-plots indicate a multi-stage crystallization with an Avrami-exponent *n* of ~3 (platelets/spherulites) at the beginning (Region I). Furthermore, a change in the slope with an exponent around 5 to 6 (complicated nucleation and spherulite form) occurs (Region II) until a high degree of crystallinity (80–95% of total crystallinity) is reached, after which the perfection of the crystalline structures takes place (*n* around 1, Region III) [33]. With increasing cooling rates, Avrami-exponents increase in all three regions and both of the first spherulite growing states become less distinctive until 8 K/min (Figure 3). At 8 K/min, Sinopec E02ES CoPo and Borealis WB140 HMS show similar values in region I and II, while this effect is less pronounced for Sinopec HMS20Z. Considering the Avrami-exponent *n* as well as the nucleation and growth rate constant *k* at the highest cooling rate, the following trends can be observed for the crystallization process of the PP grades.

- Sinopec E02ES CoPo and Borealis WB140 HMS crystallize (main crystallization area) in a very similar manner and crystallize faster than Sinopec HMS20Z at low cooling rates.
- At higher cooling rates, the most rapid main crystallization occurs for Sinopec E02ES CoPo, followed by Sinopec HMS20Z, and the slowest is Borealis WB140 HMS.
- The perfection of crystalline structures (Region III) takes significantly longer for Sinopec HMS20Z, than for Borealis WB140 HMS or Sinopec E02ES CoPo (fastest).

The complete data is summarized in Table 3.

### 3.2. Rheological Characterization

#### 3.2.1. Shear-Rheological Non-Isothermal Multiwave Measurements

Usually, the determination of cross-linking due to crystallization is qualified by the gel point. As the crystallization of polymers is a sol–gel transition of polymeric materials, a network point-like behavior of the crystals can be assumed. For the standard evaluation, the gel point is defined as crossover point of storage and loss modulus at a fixed angular frequency. The main disadvantage of this method is the dependency of the crossover on the frequency, meaning this method is only an approximation. A multiwave test, on the other hand, is performed with many frequencies simultaneously in superposition (consisting of multiple harmonics based on the fundamental wave). The material answers for each frequency is separated by a Fourier transformation. Compared to the previously mentioned method, here the loss factor tan (δ) is frequency independent at the gel point. It is then a point of intersection of all frequencies [34,35,36].

**Figure 4:**
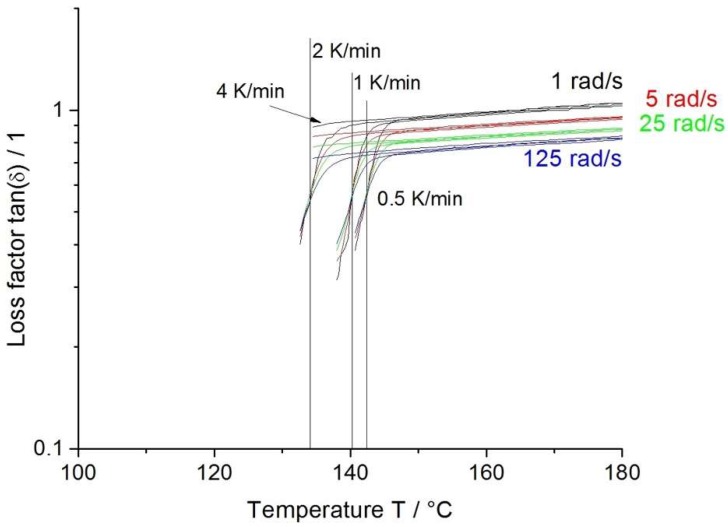
Non-isothermal crystallization multiwave measurement of Borealis WB140 HMS.

First, the Borealis WB140 HMS benchmark material was investigated. Starting from the molten polymer state, the material was cooled down at 0.5, 1, 2, and 4 K/min with different frequencies. As shown in Figure 4, the crystallization starts at ~146 °C at a cooling rate of 0.5 K/min (onset of curve drop) and hits the gel point at ~142 °C. This temperature range (cooling rate dependent) represents the foam processing window for a semicrystalline polymer, as the crystallization kinetics can strongly influence the foaming process, e.g., melt freezing in the die, cell rupture due to local freezing in a cell wall, and inhibited foam expansion (decreased elasticity). 

**Figure 5:**
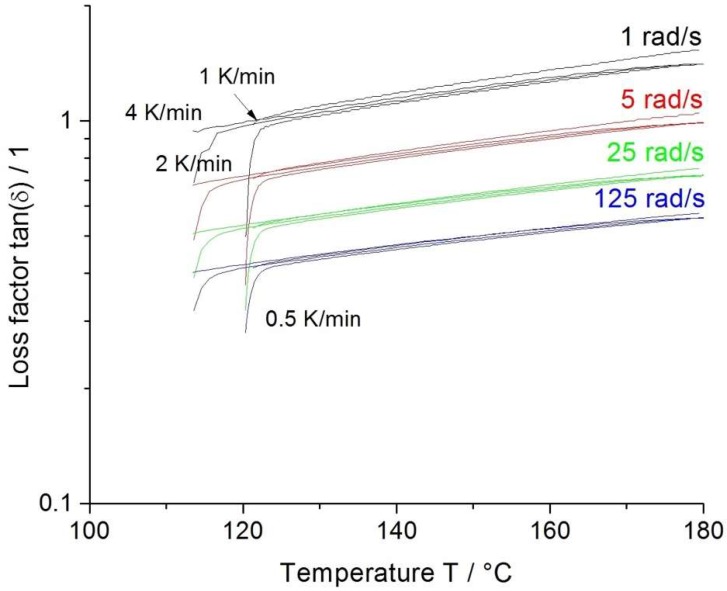
Non-isothermal crystallization multiwave measurement of Sinopec E02ES CoPo.

Compared to the benchmark material, the Sinopec E02ES CoPo behaves clearly different. The crystallization onset lies, cooling rate dependent, at lower temperatures (~12 °C) due to the PE-content, which also hinders the crystallization. When the gel point is reached the copolymer freezes immediately, showing a very narrow temperature window for processing (Figure 5). Cooling rates above 4 K/min illustrate the fast crystallization, as the valid measurement area is very limited (beginning of background noise).

**Figure 6:**
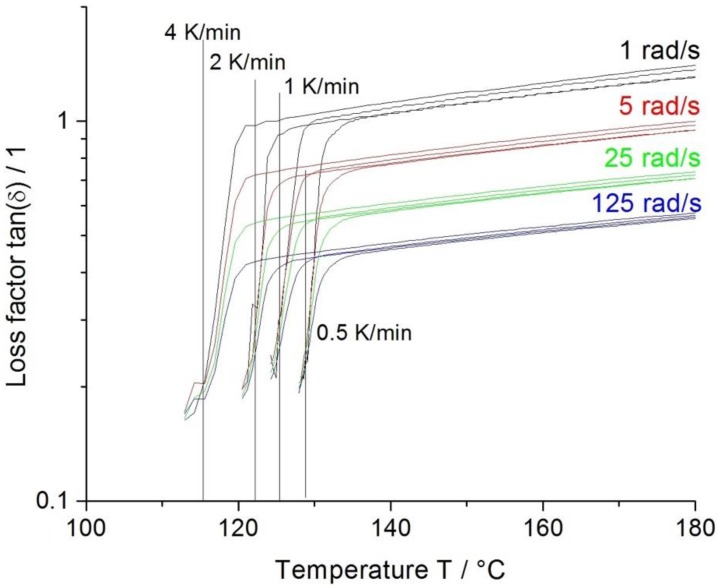
Non-isothermal crystallization multiwave measurement of Sinopec HMS20Z. The vertical line indicates the gel point.

The Sinopec HMS20Z PP also exhibits an onset point at lower temperatures, compared to the Borealis long-chain branched material, namely, at ~136 °C (Figure 6). The most noteworthy observation is that Sinopec HMS20Z solidifies gradually with a comparably broad temperature window to LCB-PP (Borealis WB140 HMS). 

Therefore, it is expected that this PP-grade will exhibit lower susceptibility to process fluctuations (more stable process) and show the most favorable crystallization behavior for foaming.

The multiwave results allow for a better understanding of the process as well as help narrow down the processing window and estimate the process stability (temperature difference between onset and gel point of the crystallization). In this frame, the Sinopec HMS20Z-PP and the Borealis WB140 HMS LCB-PP are expected to perform better in foam extrusion in terms of processing window and foam quality. 

#### 3.2.2. Isothermal Multiwave Measurements

**Figure 7:**
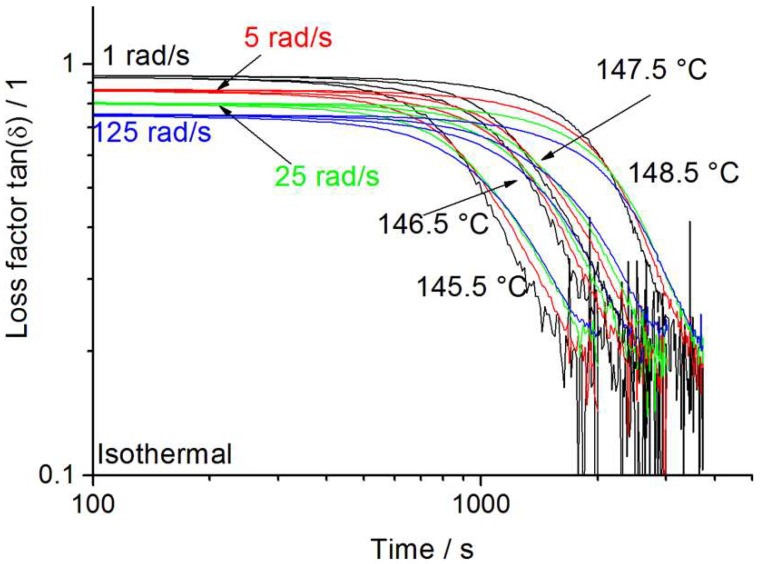
Isothermal crystallization multiwave measurement of Borealis WB140HMS.

The crystallization behavior was further analyzed with isothermal multiwave measurements. The required temperatures for this measurement were obtained around the DSC-Avrami evaluation range. For better comparability, only the temperatures closest to the Avrami range at 1 rad/s (due to similar temperature ranges for crystallization) will be discussed. As shown in Figure 7, the Borealis WB140 HMS-PP starts to crystallize after 432 s at 145.5 °C (onset after 5 % loss in tan (δ)) and remains elastic after passing through the gel point for a longer period of time (984 s) until measurement becomes invalid due to noise (after 1884 s). This indicates that even after the immobilization through the gelation, some of the elastic behavior is retained for additional 984 s (1 rad/s), making it an advantageous feature for foaming. At higher measured temperatures, Borealis WB140 HMS crystallizes significantly slower. Crystallization starts after 864 s, hits the gel point at 2213 s measuring time, and retains elasticity for further 979 s (1 rad/s). Interestingly, the times for retained elasticity are more or less similar at all investigated temperatures for this material grade.

**Figure 8:**
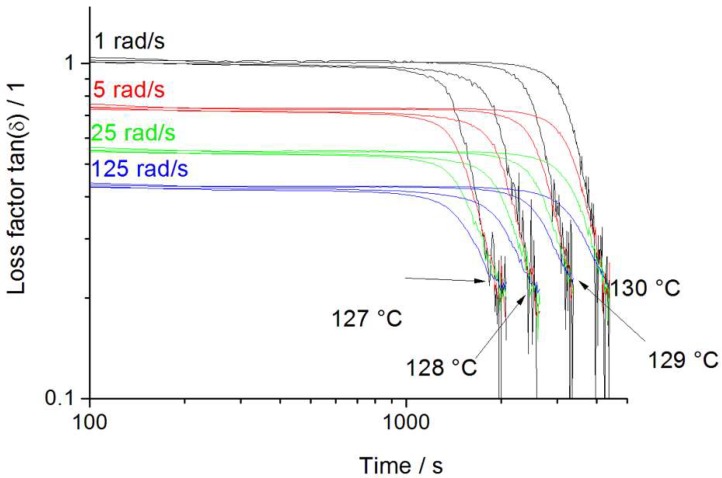
Isothermal crystallization multiwave measurement of Sinopec E02ES CoPo.

Compared to that, the crystallization process for the Sinopec E02ES CoPo PP (Figure 8) starts later after 846 s (5% loss in tan δ) at a temperature of 127 °C and reaches the gel point at 1908 s (1 rad/s). The observed times for further elastic behavior after the gel point are very short, compared to other grades. Thus, the fast crystallization process (abrupt change) can be validated again as well as the high material rigidity, as short remaining elasticity (unusual termination) is shown and noise starts almost directly after the gel point due to low deformability.

**Figure 9:**
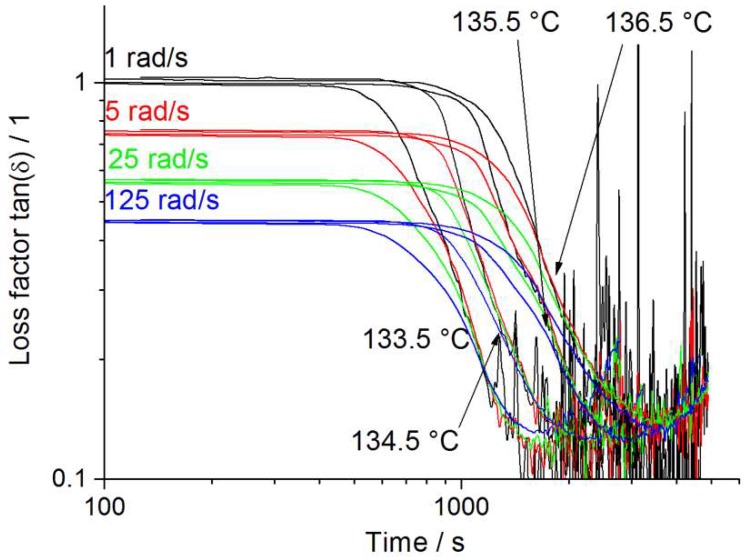
Isothermal crystallization multiwave measurement of Sinopec HMS20Z.

In Figure 9, isothermal multiwave results for the Sinopec HMS20Z PP are shown. It can be seen that this grade starts to crystallize after 756 s (onset), pacing the gel point at 2088 s and showing measurable values even for 2106 s after gelation. Consequentially, this PP-grade shows the longest retained overall elasticity of 4194 s, compared to Sinopec E02ES CoPo (elasticity for 2052 s) and Borealis WB140 HMS-PP (1884 s) and can be correlated to the crystallization process. Further data is summarized in Table 4. The difference in isothermal crystallization process between Borealis WB 140 HMS and Sinopec HMS 20Z arises from the different response of the chain topology to shear thinning, making it easier for linear chains to be disentangled (more susceptible to shearing). Retaining elasticity after the gel point, combined with slow crystallization, enables a broader processing window for foaming, thus it is possible to estimate foaming performance again. 

The findings and tendencies of the isothermal and non-isothermal multiwave measurements can be correlated very well with the Avrami–Jeziorny analysis. The fast crystallization process of Sinopec E02ES CoPo can be observed in the conversion and Jeziorny plots (fast overall crystallization also represented in *n* and *k*-values), as well as in the non-isothermal multiwave showing an abrupt termination of the measured curves after reaching the T_C_. Additionally, same tendencies are confirmed by isothermal multiwave. 

According to the DSC-evaluation, Borealis WB140 HMS has a slower crystallization process compared to Sinopec HMS 20Z-PP. The results of the multiwave, non-isothermal as well as isothermal, contradict that, as Borealis WB140 HMS crystallizes faster in isothermal measurements compared to Sinopec HMS20Z. The reason for this can be the chosen temperatures from the DSC-evaluations and should be determined through rheology in future. In general, Borealis WB140 HMS and Sinopec HMS20Z show similar behavior, and therefore a similar foaming potential can be expected.

#### 3.2.3. Elongational Rheology

As already mentioned, the elongational properties of the polymer are very important for foaming and controlling the foam morphology [14]. One of the main contributing factors for good foams usually stated in literature is strain hardening [13,37,38]. Strain hardening enables self-healing (thinner regions of cells require more energy to be stretched than thick ones, thus preventing cell coalescence and to widening processing window), which is the easiest way to ensure low-density foams [13,15,16,17,18,19]. However, this research shows that strain hardening is not a necessity to obtain good foams in continuous foaming and can be compensated material-wise by control of crystallization behavior [39].

##### Rheotens Test Result

**Figure 10:**
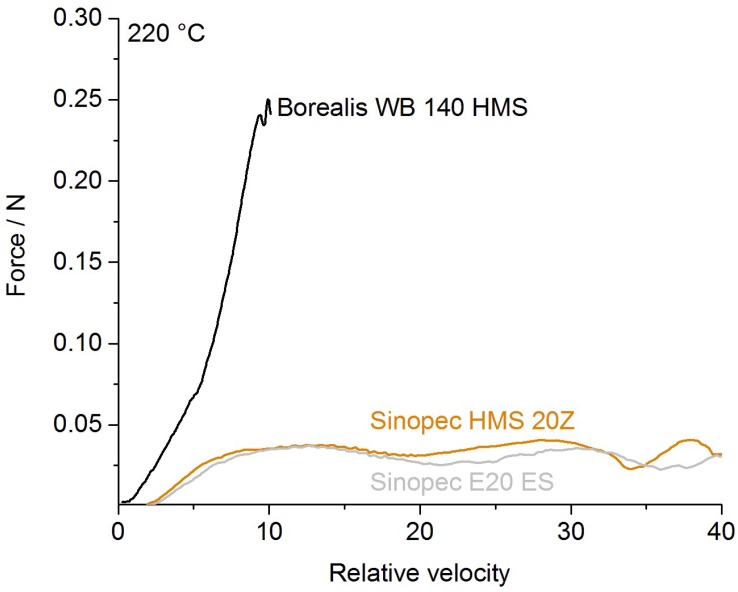
Melt drawability and strength for Borealis WB140 HMS, Sinopec E02ES CoPo, and Sinopec HMS20Z.

The drawability and melt strength of the investigated PP-grades were analyzed to get an estimate for the material’s behavior during bubble expansion. As shown in Figure 10, Borealis WB140 HMS has the highest melt strength (MS) of all tested PP grades with 0.25 N, but a rather low drawability of the melt. The strong increase in MS is caused by the branched structure in the polymer backbone, which enables short-term physical network points due to entanglements during stress. For Sinopec HMS20Z-PP, high melt strength is achieved by a different approach of combining linear polymer chains of different lengths into a grade (broad PD) according to the manufacturer. Therefore, the short-term networking, caused by branching, is missing, and both Sinopec-grades exhibit a lower melt strength at ~0.035 N but a significantly higher drawability compared to the Borealis grade.

##### Universal Extensional Fixture

**Figure 11:**
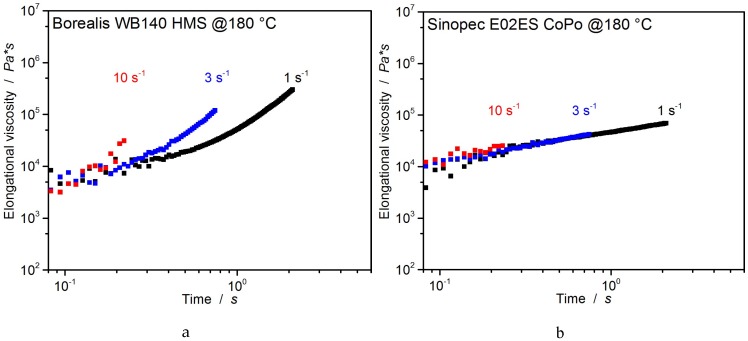
Elongation viscosity of Borealis WB140 HMS (**a**) and Sinopec E02ES CoPo (**b**) at 180 °C.

**Figure 12:**
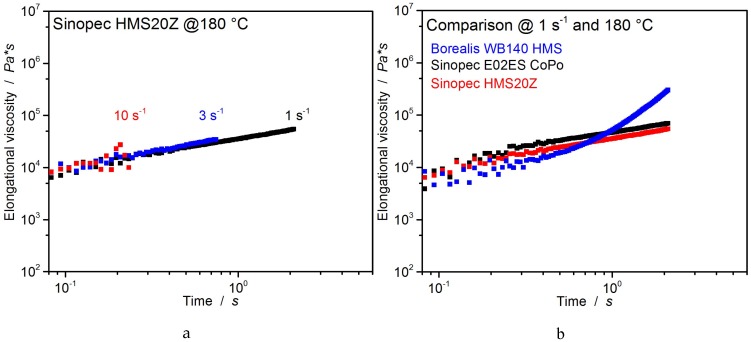
Elongation viscosity of Sinopec HMS20Z (**a**) and a comparison of all PP (**b**) at 180 °C.

For a better illustration of the transient elongational viscosities, the investigated PP-grades are compared in Figure 11 and Figure 12. The Borealis WB140 HMS-PP shows a clear strain hardening behavior at all tested strain rates. This effect is caused by the long-chain branching in the polymer structure and enables the “self-healing effect”.

This means that thin cell walls being exposed to higher strain rates, and thereby a higher chain-stretching degree, undergo an increase in elongational viscosity making thick sections easier to extend. This contributes to a more homogeneous morphology. The advantage for foaming is the preventing of cell rupture as well as coalescence through viscosity increase [6]. Sinopec E02ES CoPo expectedly exhibits no strain hardening at all strain rates investigated due to the linear nature of the polymer chains. 

Sinopec HMS20Z homo-PP behaves similarly to Sinopec E02ES CoPo, showing no strain hardening at all rates. The course of the curves and the values are very similar (Figure 12), indicating similar polymer basis. The comparison of elongational properties at a strain rate of 1 s^−1^ illustrates the similarity in performance of both Sinopec PP-grades at elongational rheology, while only Borealis WB140 HMS exhibits strain hardening (Figure 12b).

As the Sinopec materials lack strain hardening at all strain rates, they are expected (based on these results) to perform worse during extrusion foaming, resulting in higher densities and inhomogeneous morphology. On the contrary, Borealis WB140 HMS is expected to outperform both Sinopec grades in density and morphology. This conclusion is based on an established and well-known fact in literature, that strain hardening is considered advantageous and contributing significantly to good foamability.

### 3.3. Foam Extrusion

A proper foaming window for a polymer usually constitutes a compromise between the temperature (energy for expansion/elastic properties) and pressure (drop rate as driving force for gas expansion). Most characterizing methods for this difficult process aim for a thorough material characterization to extract material characteristics and thus obtain a qualitative understanding of the processing window and resulting foam morphology, as many influencing factors cannot be taken into account (e.g., high shearing rates in extruder, which cause (locally) increased melt temperatures, cannot be reproduced in a plate-plate setup in a rotational rheometer). In this frame, the obtained multiwave results (material characteristics) help narrow down the actual foaming window, as the estimations from the non-isothermal and isothermal multiwave are transferable to the actual process. For foam extrusion, the three parameters, CO_2_ content, cCO_2_; die temperature, T_Die_; and, to a certain degree, the temperature of the polymer melt, T_Melt_, were systematically studied. The zone temperatures in the A-extruder were adjusted to 180–200 °C with a pressure of 45–140 bar and in the B-extruder to 155–160 °C with an average pressure at the die around 81–95 bar. In some cases, when no satisfactory foams yielded (as for example for the Borealis WB140 HMS Material with 6% CO_2_), no results are reported.

#### 3.3.1. Variation of Blowing Agent Concentration

**Figure 13:**
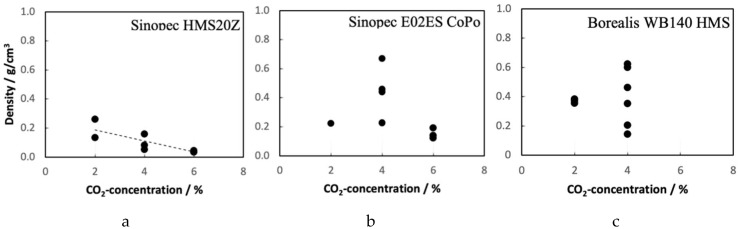
Summary of the foaming trials in terms of density as function of CO_2_ concentration for (**a**) Sinopec HMS20Z, (**b**) Sinopec E02ES CoPo, and (**c**) Borealis WB140 HMS. Please note that not only the CO_2_ concentration was varied, but also die temperature, melt temperature, and throughput.

Three different CO_2_ concentrations (2, 4, and 6 wt.%) were studied regarding their foaming potential. The resulting foam densities of the materials from these trials are shown in Figure 13. Generally, it is observed, that with increasing CO_2_ concentration, the potential for expansion increases. From the variation in density, especially for the Sinopec E02ES CoPo and the Borealis WB140 HMS polymer, it can be deducted that all processing conditions have to be well controlled to achieve a high expansion ratio. In contrast, the Sinope HMS20Z-PP delivers consistently low densities over a wide range of CO_2_ concentrations. Unfortunately, the same conditions as for the Sinopec material, were not possible for Borealis WB140 HMS because the die pressure was too high (140 bar) and fluctuating. Thus, the screw speed in the B-extruder was reduced to 9 rpm. Furthermore, it was not possible to dissolve and keep more than 4% CO_2_ at these conditions in WB140HMS; otherwise, gas leakage at the die resulted.

Densities ranging between 120 and 600 kg/m^3^ are observed for Sinopec E02ES CoPo, whereas the Sinopec HMS20Z-PP shows densities ranging between 35 and 260 kg/m^3^. Interestingly, and unexpectedly, the Borealis WB140 HMS-PP exhibits the worst foaming behavior with densities between 140 and 620 kg/m^3^. Possible explanations are given below in the section for the summary of the extrusion trials. 

#### 3.3.2. Variation of Die Temperature

**Figure 14:**
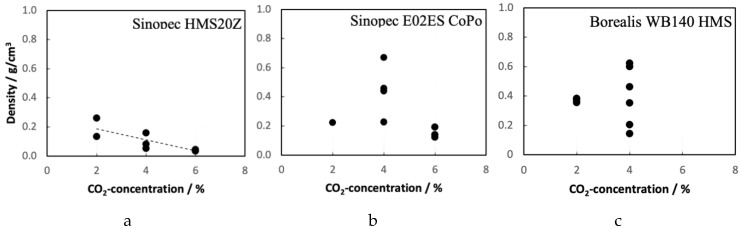
Effect of the die temperature on the foamability/density of the three PP grades for (**a**) Sinopec HMS20Z @ 6 wt.% CO_2_ & T_Melt_ = 162 °C; (**b**) Sinopec E02ES CoPo @ 6 wt.% CO_2_ & T_Melt_ = 156 °C; and (**c**) Borealis WB140 HMS @ 4 wt.% CO_2_ & T_Melt_ = 160 °C.

The die temperature is a suitable parameter to control the foam expansion and obtain information about the width of the processing window. To test the variability in foam density with the die temperature, it was varied in a range between 150 and 190 °C (when possible). The results are plotted in Figure 14. It is striking, that both Sinopec E02ES CoPo and HMS20Z exhibit a rather broad processing window as the density remains almost constant as function of die temperature. In contrast, the foamability of the Borealis WB140 HMS PP strongly depends on the die temperature. Only within a narrow range of ~10 °C (165–175 °C), good foamability is observed for this material. The Sinopec HMS20Z-PP shows only minor variations with temperature, consistently low densities between 45 and 32 kg/m^3^ are observed over the studied temperature range. When comparing the linear PPs and the LCB-PP, it can be observed that a much lower melt temperature is required for a low-density PP-foam. It is hypothesized, that the introduction of long-chain branching (as found for the Borealis type) leads to higher melt temperatures during processing due to additional viscous dissipation. Therefore, the branched polymer structure makes it difficult to achieve the required lower foaming temperatures and cooling efficiency.

#### 3.3.3. Resulting Morphology

**Figure 15:**
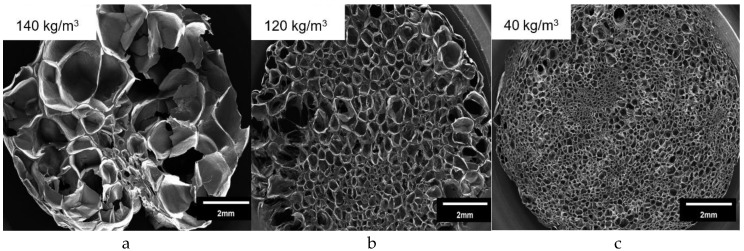
SEM-Images of Borealis WB140 HMS (**a**), Sinopec E02ES CoPo (**b**), and HMS20Z (**c**).

The morphology of the foamed strands (produced at best foaming conditions) was further investigated with SEM. The resulting SEM-micrographs are shown in Figure 15. The Borealis WB140 HMS PP-grade reaches a density of 140 kg/m^3^ with an overall coarse foam morphology (Figure 15a). It shows only a few foamed cells (cell density of 2.3*10^6^ cells/cm^2^ calculated according to work in [39]) with a bimodal cell size distribution, containing large (> 1 mm) and small cells (no homogeneous foam structure) with an average cell diameter of 0.9 mm. Borealis WB140 HMS exhibits inferior properties during foam extrusion and has not the desired foam structure, but is rather a porous solid with randomly and inhomogeneously distributed cells. By contrast, lower densities as well as smaller and more uniform cells can be achieved with Sinopec E02ES CoPo. At the lowest density of 120 kg/m^3^, a cell density of 1.7*10^7^ cells/cm^2^ and a mean cell diameter of 0.4 mm were achieved with clearly narrower size distribution (Figure 15b). Sinopec E02ES CoPo consists of mainly PP with a low percentage of PE units in the polymer backbone, making crystallization and nucleation easier due to the linear character, but is also negatively influenced by the PE-units. Therefore, a lot of cell rupture can be observed, as well as cracks in the cell walls (open cellular structure) at higher magnification. Sinopec HMS20Z-PP shows the best foamability with the lowest density of 40 kg/m^3^, a cell density of 1.6*10^8^ cells/cm^2^ and a rather fine cellular morphology, compared to other two grades, with an average cell diameter of 0.19 mm (Figure 15c). Nevertheless, a bimodal cell distribution can be observed (with very small cells in the core), which could be controlled by adding nucleating agents. As Sinopec HMS20Z only contains linear PP chains of different lengths, it exhibits the highest cell nucleation density, compared to Borealis WB140 HMS. One of the contributing factors is the presence of spherulites (correlates with degree of crystallinity) that can enhance the amount of cell nucleation sites during foaming and contributes to a finer foam morphology. During a continuous process, the melt is usually undercooled due to plasticizing effect of the blowing agent, thus the melt nearest to the barrel start to crystallize earlier. Through shearing, these crystals are mixed into the melt phase increasing viscosity and contributing as nucleating sites. When the amorphous phase crystallizes it expels the CO_2_ from crystalline regions. Therefore, locally increased blowing agent concentrations yield around spherulites (heterogeneous nucleation at amorphous/crystalline interfaces), leading to an increase in bubble nucleation [5,28,40]. This relationship was already reported and discussed in literature. Sinopec E02ES CoPo also has an increased cell nucleation density due to possible nucleation at the PP-PE interface, despite having a lower crystallinity than Borealis WB140 HMS, confirming that several factors influence the overall foam nucleation process. This can be further improved by adding nucleating agents. Once again, Borealis WB140 HMS proved to be unsuitable for continuous foaming due to the resulting bad morphology, despite expressing a strain hardening behavior.

#### 3.3.4. Correlation of Previous Findings to Foam Extrusion Trials

When comparing the three studied materials, the Sinopec HMS PP shows superior foaming characteristics, as can be seen from the lowest achieved density in Table 5 with the corresponding foaming conditions. The material also had the broadest foaming window thus guaranteeing a stable and reproducible process.

Now the question arises, where this behavior comes from. To shed some light into this fact, the Sinopec HMS-PP is compared with its competitors regarding their properties. The bubble growth at the exit of the die is mainly controlled by the viscosity, making the rheological and crystallization analysis a powerful tool for estimating foaming performance.

The crystallization behavior of Borealis WB140 HMS is predestined for discontinuous foaming and is therefore quite different compared with Sinopec HMS20Z and E02ES CoPo, with tacticity not being taken into account. The Borealis WB140 HMS-PP has a significantly higher crystallization temperature (caused by long-chain branching), which leads to lower melt expansion after the die due to earlier freezing (linear Sinopec grades do not freeze that fast). Thus, no high expansion ratios can be achieved and lower crystallization temperatures (like Sinopec Material) appear beneficial.

However, the isothermal multiwave measurements are more revealing than non-isothermal measurements, therefore making the assumptions on the material foam performance more accurate and fitting. Here, the times between start of the crystallization and the gel-point are considered. At temperatures, closest to the Avrami temperature range (DSC), the Borealis WB140 HMS PP-grade exhibits the shortest time until gelation is reached, which can be correlated to difficult processability as well as premature freezing (lower density reduction) and supports the DSC results. Thus, the Borealis WB140 HMS is rather unsuitable for continuous foaming as pure material. Blending with a linear PP-grade should be considered to counter the disadvantages as described in [29,38,39], also taking into account that this grade was designed for discontinuous foaming in the first place.

On the other hand, the linear Sinopec PP-grades have lower crystallization temperatures, which enable higher expansion ration and less premature die freezing. Additionally, the time from the onset to the gelation point of the linear PPs is 2–3-fold higher compared to Borealis WB140 HMS, which results in increased foaming performance. The enhanced foamability of Sinopec HMS20Z (compared to Sinopec E02ES) is attributed to the significantly prolonged elasticity after the gel point, which is enabled by the high PDI of 11.

In summary, a combination of low crystallization temperature and longer period of time until polymer-gelation appears to be one major key for good foamability. In contrast, “high melt strength” is only one part of the puzzle of a good foaming material and contributes significantly less in continuous foaming.

## 4. Conclusions

Polypropylene (PP) is an outstanding choice as matrix material for polymeric foams due to its favorable mechanical properties and chemical resistance. However, its semicrystalline nature and low melt elasticity result in unfavorable foaming properties. Long-chain branching of polypropylene is regarded as a game changer in foaming this material due to the introduction of strain hardening, which stabilizes the foam morphology. However, the question arises, whether it is the singular factor for obtaining low-density foams? Therefore, three PPs dedicated for foaming are studied regarding the dependency of their foaming behavior on the rheological and crystallization properties. In this frame, a linear PP Sinopec HMS20Z, a PP/PE-block co-polymer Sinopec E02ES CoPo and a long-chain branched PP Borealis WB140 HMS are the polymers of interest.

Although only the LCB-PP exhibits strain hardening and has five times the melt strength of the other grades, it does not provide the broadest foaming window or the best foam quality in terms of density (140 g/L) and cellular morphology. On the contrary, the linear Sinopec HMS20Z delivers low densities (<40 g/L) and superior foam morphology. The Sinopec E02ES CoPo performs rather negatively in terms of density and mediocre in terms of cellular morphology.

The beneficial foaming behavior of the Sinopec HMS20Z-PP is attributed to slower crystallization and a low crystallization temperature compared to the other two materials. Multiwave experiments were conducted to study the gelation due to crystallization. In isothermal multiwave experiments, the Sinopec HMS20Z exhibited a gel-like behavior over a broad time frame, whereas the other two PPs froze quickly. Non-isothermal multiwave tests also underline the finding of a broad processing window for the linear PP (Sinopec HMS20Z) as the temperature (and time) difference between the onset of crystallization and the gel point is significantly broader compared to the other PP-grades. These findings were also confirmed in DSC experiments, as especially the crystal-perfection occurs notably slower for Sinopec HMS20Z, which in turn leads to a longer gel-like state before solidification. Again, it is noted that this PP-grade exhibits no strain hardening. Thus, it is concluded that, besides sufficient rheological properties, the crystallization behavior is of utmost importance for foaming in terms of a broad processing window, when aiming to obtain low-densities and good foam morphology. In detail, a broad temperature window between the onset of crystallization and gelation are preferred for cooling, for isothermal processes an extended time in gel-like state appears beneficial.

## Figures and Tables

**Table 1:** Detailed isothermal multiwave measuring procedure/parameters for Sinopec HMS20Z.

Procedure	Step (I)	Step (II)	Step (III)
Measuring Time	13 min	2,4 min	180 min
τ	25–40 Pa	50 Pa	50 Pa
ω	1 rad/s	1 rad/s	1 rad/s
T	200 → T_measure_ + 1	T_measure_ + 1 → T_measure_	T_measure_
F_N_	0 N	0 N	0 N

**Table 2:** Crystallinity, half-time crystallization, and Avrami-temperature range for PP grades.

Sinopec HMS20Z	Crystallinity χ	t_1/2_	(t_1/2_)^−1^	Avrami Range
−2 K/min	55 %	241.8 s	4.14 × 10^−3^ s^−1^	134–106 °C
−4 K/min	54 %	159 s	6.29 × 10^−3^ s^−1^	133–98 °C
−8 K/min	54 %	103.8 s	9.63 × 10^−3^ s^−1^	132–90 °C
−16 K/min	52 %	83.4 s	11.99 × 10^−3^ s^−1^	136–74 °C
Sinopec E02ES CoPo				
−2 K/min	39 %	235.2 s	4.25 × 10^−3^ s^−1^	124–99 °C
−4 K/min	40 %	148.8 s	6.72 × 10^−3^ s^−1^	122–92 °C
−8 K/min	39 %	103.2 s	9.69 × 10^−3^ s^−1^	122–86 °C
−16 K/min	41 %	83.4 s	11.99 × 10^−3^ s^−1^	123–75 °C
Borealis WB140 HMS				
−2 K/min	44 %	354 s	2.82 × 10^−3^ s^−1^	143–112 °C
−4 K/min	44 %	215.4 s	4.64 × 10^−3^ s^−1^	142–99 °C
−8 K/min	44 %	137.4 s	7.28 × 10^−3^ s^−1^	143–94 °C
−16 K/min	39 %	87.6 s	11.42 × 10^−3^ s^−1^	145–85 °C

**Table 3:** Avrami-exponent *n*, nucleation, and growth rate constant *k* at the different cooling rates.

	n Region I	n Region II	n Region III	k I / k II / k III
Sinopec HMS20Z				
−2 K/min	2.9	5.0	0.9	2.0 / 1.5 / 0.5
−4 K/min	3.5	6.4	1.0	1.7 / 1.0 / 0.1
−8 K/min	4.6	8.1	1.1	1.0 / 0.6 / −0.2
−16 K/min	6.4	10.7	1.2	0.6 / 0.3 / −0.3
Sinopec E02ES CoPo				
−2 K/min	3.2	5.9	1.1	1.9 / 1.3 / 0.7
−4 K/min	4.9	6.8	1.2	1.3 / 1.0 / 0.4
−8 K/min	10.7	9.8	1.4	0.6 / 0.6 / 0.2
−16 K/min	11.7	11.3	1.6	0.4 / 0.3 / −0.1
Borealis WB140 HMS				
−2 K/min	3.1	5.8	1.3	2.3 / 1.8 / 1.3
−4 K/min	4.1	6.9	1.1	1.7 / 1.3 / 0.8
−8 K/min	7.3	7.1	1.5	0.9 / 0.9 / 0.5
−16 K/min	9.2	8.4	1.5	0.4 / 0.4 / 0.1

**Table 4:** Supplementary data for isothermal multiwave investigation of PP grades at 1 rad/s.

	Cryst. Start at	Gel-Point at	Onset => Gel-Point	Elastic after GP for
Sinopec HMS20Z				
133.5 °C	486	1170 s	684 s	1206 s
134.5 °C	648 s	1548 s	900 s	1548 s
**135.5 °C**	**756 s**	**2088 s**	**1332 s**	**2106 s**
136.5 °C	846 s	2592 s	1746 s	2034 s
Sinopec E02ES CoPo				
**127 °C**	**846 s**	**1908 s**	**1062 s**	**144 s**
128 °C	990 s	2412 s	1422 s	234 s
129 °C	1584 s	3289 s	1705 s	76 s
130 °C	2214 s	4176 s	1962 s	219 s
Borealis WB140 HMS				
**145.5 °C**	**432 s**	**900 s**	**468**	**984 s**
146.5 °C	522 s	1310 s	788	979 s
147.5 °C	684 s	1377 s	693	1112 s
148.5 °C	864 s	2213 s	1349	979 s

Note: highlighted temperatures resemble nearest data sets of multiwave to avrami-ranges.

**Table 5:** Best performing settings for foam extrusion with lowest densities.

	Sinopec E02ES CoPo	Sinopec HMS20Z	Borealis WB140 HMS
CO_2_ / %	6	6	4
T_melt_ / °C	156	165	164
T_die_ / °C	156	190	170
P_die_ / bar	95	81	43
Density / kg/m^3^	121	34	143
